# *PI3KCA* Mutations in Uterine Cervix Carcinoma

**DOI:** 10.3390/jcm10020220

**Published:** 2021-01-10

**Authors:** Ioannis A. Voutsadakis

**Affiliations:** 1 Algoma District Cancer Program, Sault Area Hospital, Sault Ste. Marie, ON P6B 0A8, Canada; ivoutsadakis@nosm.ca; 2 Section of Internal Medicine, Division of Clinical Sciences, Northern Ontario School of Medicine, Sudbury, ON P3E 2C6, Canada

**Keywords:** cervical cancer, squamous, mutations, PI3K kinase, catalytic subunit, *PIK3CA* mutations

## Abstract

Background: Squamous cervical carcinoma represents an infection-associated malignancy that produces a high mortality when metastatic or recurrent after primary local treatment. There is an urgent need for new therapies for this cancer. Molecular lesions in cervical cancer may provide opportunities for targeted therapies development. Methods: Publicly available data from the Cancer Genome Atlas (TCGA) were analyzed to define the molecular landscape of squamous cervical carcinomas with and without mutations of *PIK3CA*, the gene encoding the alpha catalytic subunit of phosphatidylinositol 3 kinase (PI3K). Associations with alterations in other critical genes and pathways of cancer and the total mutation burden and copy number alteration burden of cervical cancers were examined. Results: Mutations in *PIK3CA* are observed in 27.1% of squamous cervical cancers. *PIK3CA* represents the most frequently mutated gene in these cancers. Mutations in *PIK3CA* are associated with higher rates of mutations in other genes of important cancer-associated pathways such as the tyrosine kinase receptors/K-Ras/BRAF/MAPK and the Wnt/β catenin pathway. In addition, *PIK3CA* mutated cervical cancers display a higher tumor mutation burden (TMB) than non-mutated cancers. Conclusion: Frequent mutations of *PIK3CA* gene in squamous cervical carcinomas may represent an opportunity for targeted therapies development both inhibiting the PI3K kinase and associated pathway defects. Increased TMB may additionally confer immunotherapy sensitivity.

## 1. Introduction

Squamous carcinoma of the uterine cervix is an infectious cancer associated with high risk sub-types of the human papilloma virus (HPV) and represents a major public health burden worldwide. Uterine cervical cancer represents the fourth most common cancer in both incidence and mortality in women and led to more than 300,000 deaths worldwide in 2018 [1]. The introduction of HPV vaccines a few years ago is expected to improve these grim statistics in the coming decades [2]. However, cervical cancer will remain a significant health problem for the coming years with an unmet need for effective therapies, especially in the metastatic setting. Molecular characterization of cervical cancer pathogenesis holds the promise for advancing therapeutics through the development of rational targeted therapies.

*PIK3CA*, the gene encoding for the alpha catalytic subunit of phosphatidylinositol-4,5-bisphosphate 3-kinase, is one of the most frequently mutated oncogenes in cancer [3]. The gene is located at the long arm of human chromosome 3 at 3q26.32. The catalytic unit encoded by *PIK3CA* consists of 1068 aminoacids, has a molecular weight of 110 kDa, and is regulated by a regulatory subunit of 85 kDa, encoded by PIK3R1. The kinase receives activating signals from receptor tyrosine kinases and functions to phosphorylate phosphatidylinositol-4,5-bisphosphate to phosphatidylinositol-3,4,5-triphosphate, which binds kinase PDK1, the kinase complex mTORC2 and the serine/threonine kinase AKT. The proximity of AKT with the two other kinases facilitates phosphorylation of AKT [4]. This phosphorylation results in activation of kinase AKT. AKT phosphorylates and activates or inhibits, in its turn, several downstream effectors with pro-carcinogenic actions. An important down-stream effector is the mTORC1 complex, whose activation results in cell growth and protein translation promotion through inhibition of kinase S6K1 and of translation negative regulator EIF4EBP1, respectively [5]. In addition, through the action of multiple downstream effectors, PI3K regulates other cancer associated pathways. For example, mTORC1 executes an inhibitory phosphorylation on kinase GSK3 (Glycogen Synthase Kinase 3), an inhibitor of the Wnt/β-catenin pathway [6]. AKT phosphorylation also leads to inhibitory phosphorylation of tumor suppressors including the transcription factor FOXO1 [7].

*PIK3CA* mutations are observed frequently in a broad spectrum of cancers, including the most prevalent cancers such as breast, endometrial, and colorectal cancers [8]. In breast cancer, *PIK3CA* mutations are present in about a third of patients and are more common in ER-positive, HER2-negative cancers [9]. The great majority of mutations are observed in hotspot codons H1047, E542, E545, and N345. *PIK3CA* mutations have come to the therapeutic forefront in metastatic ER-positive, HER2-negative breast cancers, as they predict response to PI3K inhibitors [10]. In this sub-type of breast cancer, the alpha-specific PI3K inhibitor alpelicib has been approved in combination with hormonal therapy in the metastatic setting after progression on CDK inhibitors combinations [11]. In colorectal adenocarcinomas, *PIK3CA* mutations occur in 20% to 25% of cases [12,13,14]. Their presence has been suggested to confer resistance to standard colorectal cancer FOLFOX (5-FU, Folinic acid, Oxaliplatin) chemotherapy [15].

This article examines the landscape of *PIK3CA* mutated cervical cancers and compares it with that of *PIK3CA* wild-type carcinomas. In the current therapeutic environment of targeted and personalized therapies, identification and characterization of specific sub-sets of cancers that used to be thought and treated as homogeneous is increasingly important. Currently, the VEGF inhibitor bevacizumab and the PD-1 inhibitor pembrolizumab are the only targeted therapies approved for metastatic cervical carcinomas. Bevacizumab indication is not biomarker based and pembrolizumab indication in the molecule PD-1 positive population provides an imperfect targeting, as the response rate is less than 15%. Additional targeted drugs with associated biomarkers would be more than welcome in metastatic cervical carcinomas. PI3K inhibitors are already in the clinic for other cancers and, given the high prevalence of *PIK3CA* mutations in the disease, as ascertained in the current study, they could be prioritized for further development in cervical carcinomas.

## 2. Materials and Methods

The Cancer Genome Atlas (TCGA) study of cervical cancer was used to compare patients with or without *PIK3CA* mutations regarding clinical and genomic characteristics of interest [16]. The study includes a total of 297 patients, among whom 46 patients have adenocarcinoma and were excluded from the current analysis. The remaining 251 patients with squamous cervical carcinoma were included in the analysis.

The analysis was performed in the cBioPortal for Cancer Genomics Portal (cBioportal, http://www.cbioportal.org), a genomics site initially developed by Memorial Sloan Kettering Cancer Center (MSKCC) and currently maintained by MSKCC in collaboration with others, which allows for a user friendly interrogation of any gene of interest and includes data on mutations, copy number alterations (CNAs), mRNA expression, as well as correlative clinical data [17]. For the analysis of CNAs in TCGA, the GISTIC (Genomic Identification of Significant Targets in Cancer) algorithm is used. A score of 2 or above in GISTIC denotes putative amplification of a gene, while a score of -2 and below denotes putative deep deletion. The Aneuploidy Score (AS) is derived by summing the number of chromosome arms in each sample that have copy number alterations (gains or losses). A chromosome arm is considered copy number altered, gained, or lost, if there is a somatic copy number alteration in more than 80% of the length of the arm, as calculated by the ABSOLUTE algorithm from Affymetrix 6.0 SNP arrays [18]. Chromosomal arms with somatic copy number alterations in 20% to 80% of the arm length are not called, and chromosomal arms with somatic copy number alterations in less than 20% of the arm length are considered not altered. The fraction of genome altered (FGA) in TCGA is derived by source segment files and is calculated by summing the length of segments with log2 greater than 0.2 divided by the total length of all segments measured in the sample. FGA represents another measure of the burden of copy number alterations in a sample. Information of the pathogenic implications of mutations in genes of interest was derived from the OncoKB knowledgebase [19].

Prognosis of squamous cervical cancer patients with or without *PIK3CA* mutations was evaluated by construction of Kaplan Meier curves. Statistical comparisons of categorical and continuous data were carried with the Fisher’s exact test or the x^2^ test and the t test. The Log Rank test was used to compare Kaplan–Meier survival curves. All statistical comparisons were considered significant if *p* < 0.05.

## 3. Results

**Figure 1:**
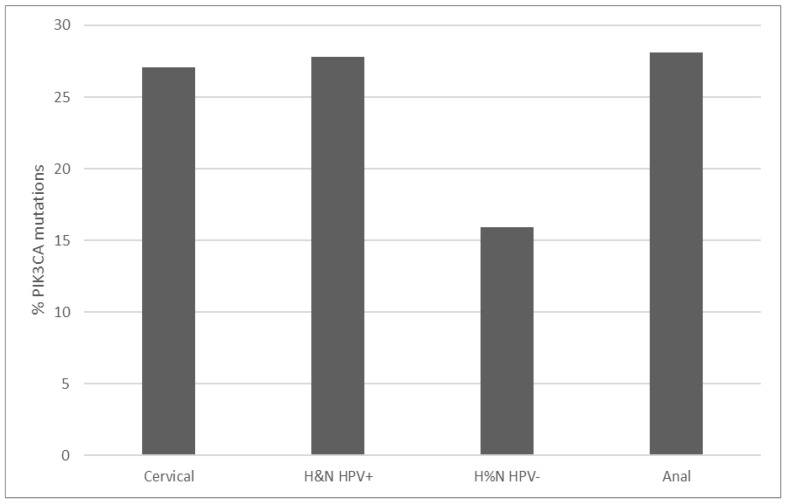
Prevalence of *PIK3CA* mutations in cervical carcinomas, HPV+ and HPV– head and neck carcinomas and anal carcinomas. Fisher’s exact test *p* = 0.01 for the comparison of *PIK3CA* mutations in HPV+ and HPV– head and neck carcinomas. Data are from TCGA and MSK-IMPACT.

*PIK3CA* mutations prevalence in TCGA cervical squamous cell carcinoma study is 27.1% (67 of 251 cases, Figure 1). Thirty-nine cases (15.5%) display amplifications of the gene located at 3q26, and a total of 39% of cases have either mutations or amplifications or both. This makes *PIK3CA* the most prevalent lesions in squamous cervical carcinomas in TCGA. *PIK3CA* mutations observed are missense mutations, and two thirds are concentrated in aminoacid positions E542 and E545 located in the helical PI3Kα family accessory domain (aminoacids 520 to 703) of the protein. These hot spot mutations are known to be oncogenic, as listed in the OncoKB cancer mutations knowledgebase. Most mutations in other aminoacid positions observed in *PIK3CA* are also listed as oncogenic or likely oncogenic in OncoKB. Consistent with their prevalence in cervical squamous cell carcinomas, *PIK3CA* mutations are observed in 27.8% and 28.1% of cases in HPV+ head and neck cancers and anal carcinomas from the head and neck TCGA study and the anal carcinoma subset of the MSK-IMPACT cohort (Figure 1) [8,20]. In contrast, HPV– head and neck cancers display a lower *PIK3CA* mutations prevalence of 15.9%.

**Figure 2:**
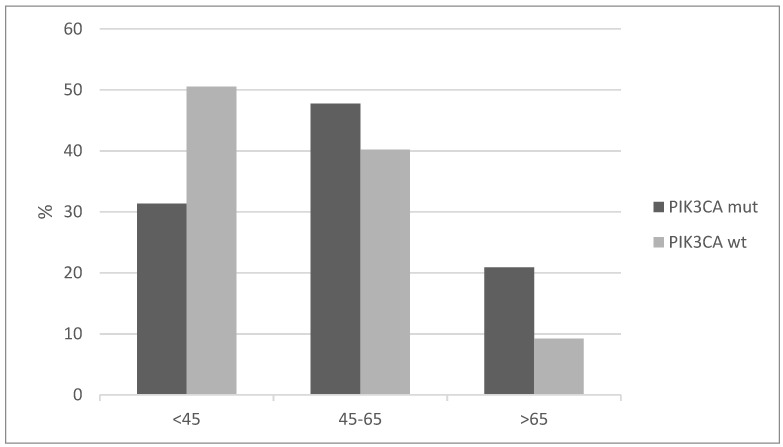
Age distribution of squamous cervical carcinoma patients with and without *PIK3CA* mutations (x^2^ *p* = 0.006).

Patients with cervical squamous carcinomas and *PIK3CA* mutations tend to present in a more advanced age than the cervical cancer patients without *PIK3CA* mutations. More than 20% of cases in the group with *PIK3CA* mutations were older than 65, while the percentage of older than 65 years-old patients in the group without *PIK3CA* mutations was less than 10% (Figure 2). Conversely, half of the patients without *PIK3CA* mutations present at age 45 and younger.

**Figure 3:**
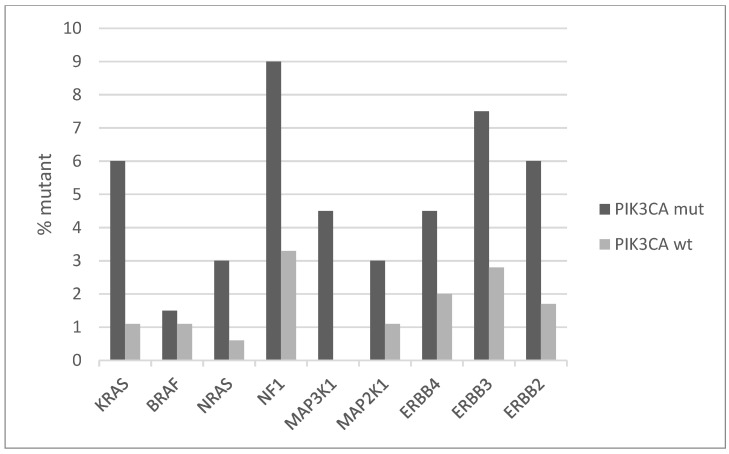
Percentage of mutations of genes of the receptor tyrosine kinase-RAS-RAF-MEK cascade in *PIK3CA* mutant and wild-type cervical cancers. Fisher’s exact test *p* = 0.002 for the comparison of cases with mutations in any of these genes versus cases with none of these genes mutated in the two groups (*PIK3CA* mutant and *PIK3CA* wild-type). Data are from TCGA.

The signaling pathway transducing signals from cell surface tyrosine kinase receptors (TKRs) through K-Ras to MAPK kinases is commonly dysregulated in cancer. Several components of this pathway show low numbers of mutations in squamous cervical cancer. The prevalence of mutations of the backbone component genes of the pathway is higher in cervical cancers with *PIK3CA* mutations compared with the group without *PIK3CA* mutations (Figure 3). Overall, the *PIK3CA* mutant group has mutations in one or more genes of the TKRs/K-Ras/MAPK pathway in 38.8% of cases, while the *PIK3CA* wild-type group has mutations in one or more genes of the TRK/K-Ras/MAPK pathway in 19% of cases (Fisher’s exact test *p* = 0.002). The most prevalent mutations are at the gene for the neurofibromin 1 (NF1), a negative regulator of K-RAS, KRAS itself, and the EGFR family TKRs ERBB3 and ERBB2, which are mutated in 9%, 6%, 7.5%, and 6% of *PIK3CA* mutated cases, respectively (Figure 3). All but one KRAS mutations are hotspot mutations and known to be oncogenic. About half of the mutations in the three other genes are known oncogenic, while the rest are currently variants of unknown significance.

**Figure 4:**
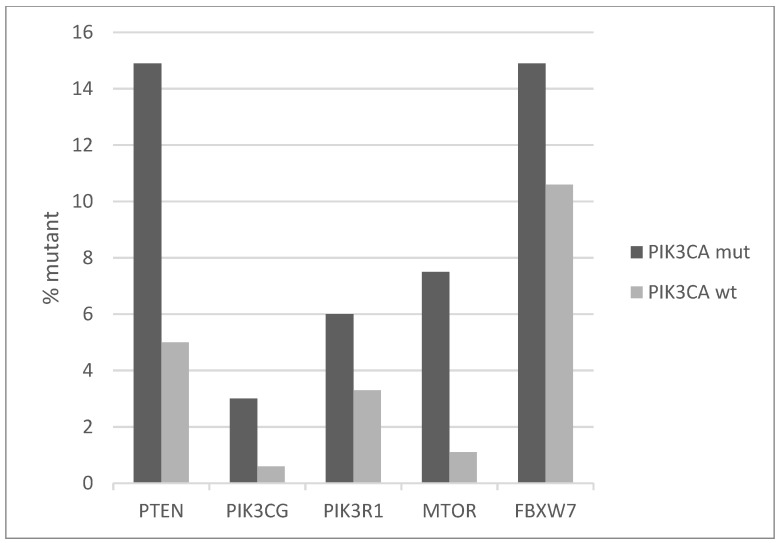
Percentage of mutations of genes of the PI3K-AKT-PTEN cascade in *PIK3CA* mutant and wild-type cervical cancers. Fisher’s exact test *p* = 0.01 for the comparison of cases with mutations in any of these genes versus cases with none of these genes mutated in the two groups (*PIK3CA* mutant and *PIK3CA* wild-type). Data are from TCGA.

The signaling cascade in which PI3K kinase belongs receives also input from TKRs in parallel with K-RAS-MAPK, through the PI3K and AKT kinases and presents increased prevalence of mutations in *PIK3CA* mutated cervical cancers compared with non-mutated counterparts (Figure 4). In the *PIK3CA* mutated group, one or more key proteins of the pathway are mutated in 34.3% of cases, and in the *PIK3CA* wild-type group, they are mutated in 22.3% of cases. The most frequently mutated genes are the tumor suppressors PTEN and FBXW7, which are both mutated in 14.9% of cases in *PIK3CA* mutated cancers and in 5% and 10.6% of cases, respectively, in *PIK3CA* wild-type cancers (Figure 4). In addition, small numbers of recurrent deep deletions are present in both PTEN and FBXW7, which increases the total prevalence of molecular lesions in these genes in cervical squamous carcinomas to 13% and 14%, respectively. Most PTEN and FBXW7 cervical carcinoma mutations are considered likely oncogenic in OncoKB database, while for a few the significance is unknown.

**Figure 5:**
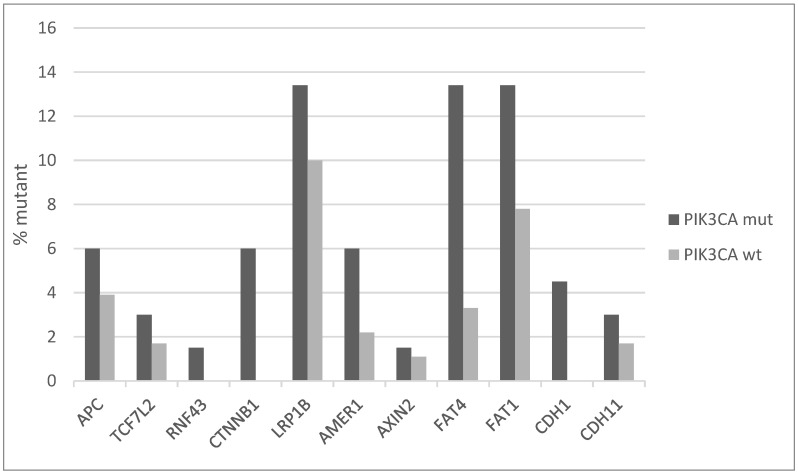
Percentage of mutations of genes of the Wnt/β catenin pathway in *PIK3CA* mutant and wild-type cervical cancers. Fisher’s exact test *p* = 0.03 for the comparison of cases with mutations in any of these genes versus cases with none of these genes mutated in the two groups (*PIK3CA* mutant and *PIK3CA* wild-type). Data are from TCGA.

The Wnt/β catenin pathway also presents an increased number of mutations in key component genes in *PIK3CA* mutated cervical cancers compared with *PIK3CA* wild-type counterparts. Most frequently mutated genes of the pathway are the co-receptor LRP1B (LDL receptor related protein 1B) and the FAT type cadherins FAT4 and FAT1, which have all a prevalence of mutations in *PIK3CA* mutated cancers of 13.4%, while in the non-mutated group they are mutated in 10%, 3.3%, and 7.8% of cases, respectively (Figure 5). The core component genes of the pathway CTNNB1, encoding for β catenin, APC and CDH1, encoding for E cadherin, are less frequently mutated, but also their prevalence is higher in *PIK3CA* mutated cancers than wild-type ones. LRP1B undergoes, in addition to mutations, deep deletions in cervical cancer for a total frequency of genetic lesions of 20%, suggesting an important tumor suppressor role.

**Figure 6:**
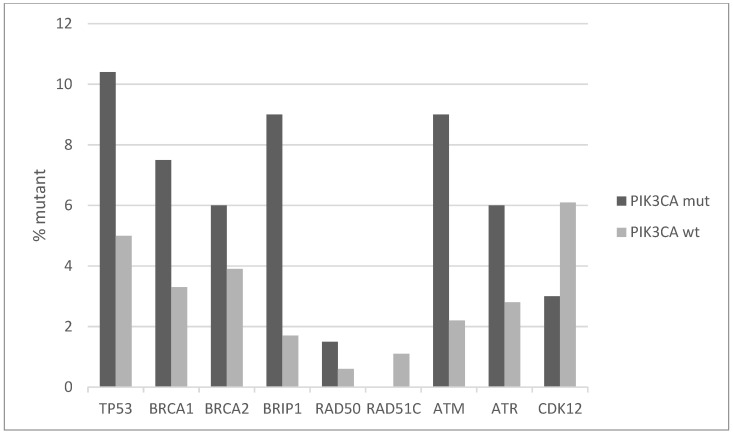
Percentage of mutations of genes involved in DNA damage response and repair pathways in *PIK3CA* mutant and wild-type cervical cancers. Fisher’s exact test *p* = 0.15 for the comparison of cases with mutations in any of these genes versus cases with none of these genes mutated in the two groups (*PIK3CA* mutant and *PIK3CA* wild-type). Data are from TCGA.

Squamous cell carcinomas of the uterine cervix possess a low rate of TP53 mutations, as the function of this seminal tumor suppressor is debilitated, instead, by HPV E6 protein. However, a small number of cervical cancers do possess TP53 mutations, and those are more common in *PIK3CA* mutated cancers (10.4% versus 5% *PIK3CA* wild-type cancers, Figure 6). Other proteins involved in DNA damage response and repair have higher mutation rates in the *PIK3CA* mutated sub-set of squamous cervical carcinomas. BRCA1, BRCA2, BRIP1, ATR, and ATM have all a mutation frequency of 6% or higher (up to 9% for BRIP1 and ATM) in *PIK3CA* mutated cancers, although the overall mutation rate in the DNA damage response genes did not reach statistical significance between the *PIK3CA* mutant and *PIK3CA* wild-type groups (Fisher’s exact test *p* = 0.15, Figure 6). Most mutations observed in these genes are currently of unknown significance.

**Figure 7:**
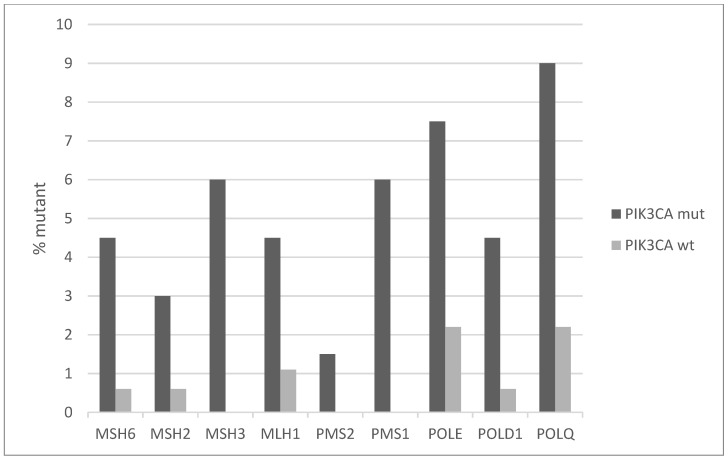
Percentage of mutations of mismatch repair (MMR) genes and genes encoding for the proof-reading polymerases epsilon and delta (POLE and POLD1) in *PIK3CA* mutant and wild-type cervical cancers. Fisher’s exact test *p* = 0.0004 for the comparison of cases with mutations in any of these genes versus cases with none of these genes mutated in the two groups (*PIK3CA* mutant and *PIK3CA* wild-type). Data are from TCGA.

**Figure 8:**
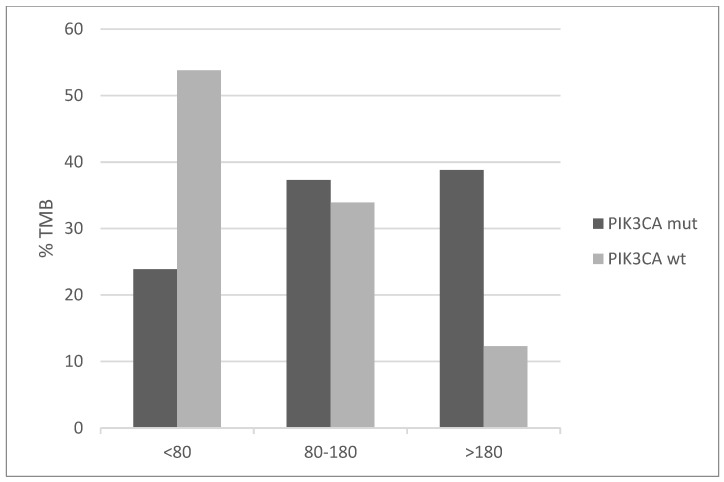
Percentage of cases with different levels of total mutation burden (TMB) in *PIK3CA* mutant and wild-type cervical cancers (x^2^ *p* < 0.001). Data are from TCGA.

Mutations of genes involved in mismatch repair (MMR) and producing microsatellite instability (MSI) are overall rare in cervical cancers with an individual frequency of 2% or less. However, the *PIK3CA* mutated group displays higher frequencies ranging from 3% to 6% (Figure 7). Similarly, the genes for proofreading polymerases POLE and POLD1 are mutated in 7.5% and 4.5% of *PIK3CA* mutated cancers versus 2.2% and 0.6% of *PIK3CA* non-mutated cancers. Consistent with the higher mutation frequency of MMR and proofreading polymerases mutations and the known association of MSI with high total mutation burden (TMB), *PIK3CA* mutated cervical cancers display a higher TMB than *PIK3CA* wild-type counterparts (Figure 8). Importantly, almost 40% of *PIK3CA* mutated cervical cancers have a TMB of above 180. This percentage is only 12.3% in unmutated cancers.

**Figure 9:**
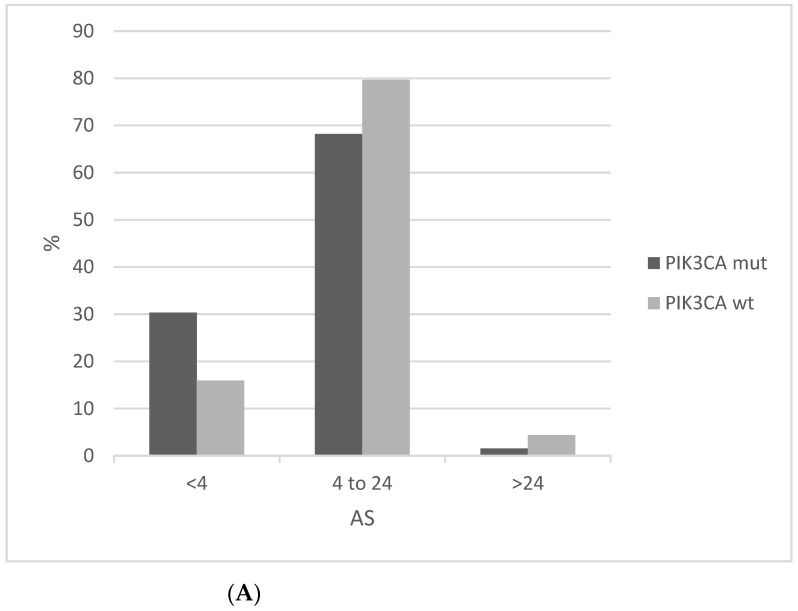

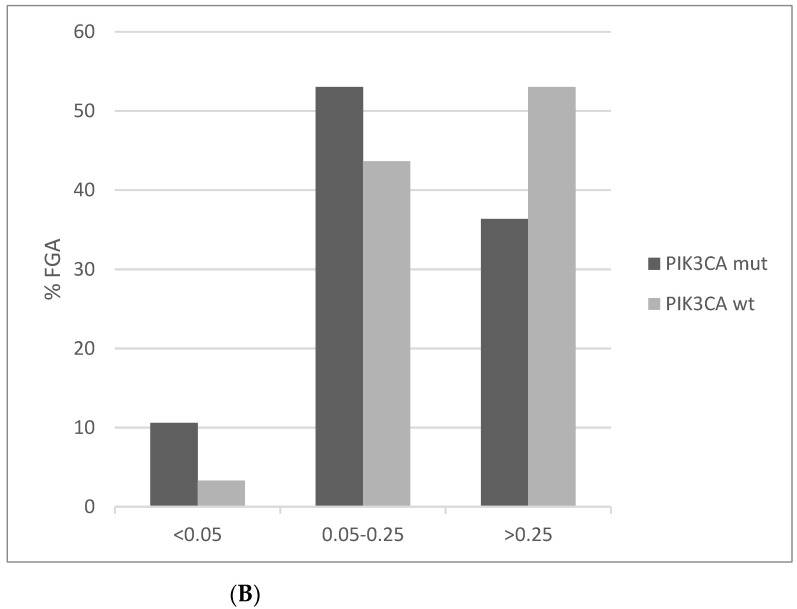
Percentage of cases with different levels of chromosomal instability as measured by (**A**) The Aneuploidy Score (AS) (x^2^ *p* = 0.03). (**B**) The fraction of genome altered (FGA) in *PIK3CA* mutant and wild-type cervical cancers (x^2^ *p* = 0.01). Data are from TCGA.

In contrast to MSI, chromosomal instability as measured by AS is lower in *PIK3CA* mutated cervical cancers compared to *PIK3CA* wild-type counterparts (Figure 9A). AS below 4 is observed in 30.3% mutated cancers compared to 15.9% of *PIK3CA* wild-type squamous cervical carcinomas. In addition, the *PIK3CA* mutated group presents a higher percentage of low Fraction of Genome Altered (FGA, another measure of chromosomal instability) cases than the *PIK3CA* wild-type group (Figure 9B).

As mentioned above, about 15% of cervical cancers bear amplifications of *PIK3CA* as part of a commonly amplified locus at 3q26-28. This locus contains also other oncogenes, including MECOM, encoding for oncogene EVI1, SOX2, the p53 family member TP63, and ETV5. The area of the long arm of chromosome 3 at 3q26-28 is commonly amplified in other squamous carcinomas such as lung and head and neck cancers. A comparison of the rate of *PIK3CA* mutations in 3q26-28 amplified and non-amplified cervical cancers disclosed no significant difference, with the amplified group harboring *PIK3CA* mutations in 26.3% of cases and the non-amplified group harboring such mutations in 27.3% of cases.

**Figure 10:**
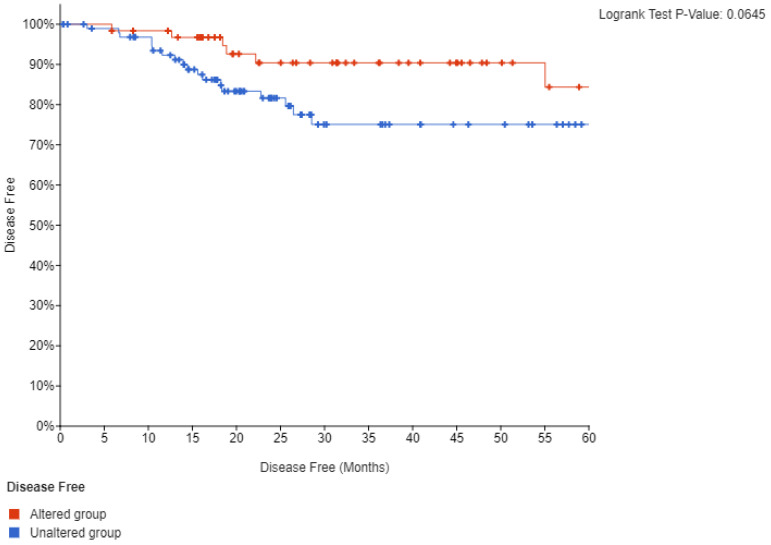
Disease free survival (DFS) of cervical cancer patients with mutations or amplifications of *PIK3CA* compared with patients with no molecular lesions in the gene.

Survival analysis of TCGA cohort disclosed that neither mutations nor amplifications of *PIK3CA* are prognostic for disease free survival (DFS) or overall survival (OS) (not shown). However, when lesions of *PIK3CA* were combined a trend towards a better DFS in the group with *PIK3CA* alterations that did not reach statistical significance (*p* = 0.06) was observed (Figure 10). OS remained not different between groups. These results suggest that despite the key role of PI3K kinase in oncogenesis, it is not prognostic for outcomes in squamous cervical carcinoma. However, the fact that DFS was worse, albeit not reaching statistical significance, in cancers with PI3K lesions is intriguing and suggests that PI3K dysfunction may have prognostic implications in these cancers. This could be confirmed with a more extensive study size.

## 4. Discussion

Squamous cervical carcinomas present a particular molecular landscape characterized by low prevalence of mutations in the tumor suppressor p53. Instead, in these carcinomas, the multifaceted functions of p53 in suppressing virtually all enabling characteristics of cancer are neutralized by the HPV protein E6 [21]. E6 promotes proteasome degradation of wild-type p53 by facilitation of ubiquitination of p53 by ubiquitin ligase UBE3A, also known as E6-AP (E6-Associated Protein) [22]. In addition, the viral protein E7 has been implicated in HPV related tumorigenesis by interference with the function of the tumor suppressor RB and neutralization of cell cycle checkpoints. As a result, cancer cell proliferation becomes independent from physiologic signals that normally regulate untimely cell cycle entry. *PIK3CA* encodes for the catalytic sub-unit of lipid kinase PI3K, which is an internal part of signaling cascades that connect receptor tyrosine kinases to the mTOR pathway and protein synthesis, inhibition of cell cycle arrest, and inhibition of apoptosis [3]. It also transmits signals through the TGFβ/Smad signaling pathway and the Wnt/β-catenin pathway, both important transducers of oncogenesis-related signals and altered in various cancers. Thus, in cervical carcinomas, *PIK3CA* activation through mutations could assist viral-induced oncogenesis by further incurring on p53 inhibition and enhancing proliferation by cell cycle promotion.

*PIK3CA* is the second most frequently mutated oncogene across cancers [23]. In the TCGA cohort of squamous cervical carcinomas, it is mutated in 24%, showing the highest prevalence of mutations of all oncogenes in these cancers. Clinically, *PIK3CA* mutations are associated with a more advanced age at presentation. This association with older age at presentation had also been observed in another series of cervical carcinomas that included both squamous and adenocarcinoma histologies [24]. The subset of cervical cancers with *PIK3CA* mutations tend to have additional mutations in cancer-associated genes of oncogenic pathways more frequently than patients without *PIK3CA* mutations. Consistent with this observation, the percentage of patients with *PIK3CA* mutations and a high TMB is higher than in patients without *PIK3CA* mutations. MMR and proofreading polymerases mutations are more often seen in *PIK3CA* mutated cancers. However, the great majority of *PIK3CA* mutations are concentrated in a few hotspot codons and almost the totality of the remaining mutations is classified as probably oncogenic, strongly suggesting that they are not passenger mutations occurring by chance in hypermutated cancers. Surprisingly, increased TMB in *PIK3CA* mutated cancers does not translate to clearly better survival outcomes, implying that any positive influence of the hypermutated phenotype may be neutralized by other factors in the *PIK3CA* mutated cellular micro-environment. In colorectal cancers, MSI are associated with a better prognosis than microsatellite stable cancers [25]. Thus, other factors present in the cervical cancer microenvironment, beyond the increased number of antigens presented to immune effectors, are at play in MSI related cancers and hinders an improved prognosis. In that respect, the oncogenic HPV-encoded E5 protein interferes with antigen presentation and attenuates immune responses [26]. In another series of mostly squamous cervical cancers, early stage IB and II *PIK3CA* mutated cancers had worse survival than patients with same stages *PIK3CA* wild-type cancers [27]. In contrast, no survival difference was observed in stage III and IVA cancers. Yet in a series with advanced and metastatic pretreated patients with squamous cervical carcinomas who had participated in phase I trials, patients with *PIK3CA* mutations had a better survival than non-mutated patients [28].

Amplifications of the locus of *PIK3CA* at chromosome arm 3q26 are the most common copy number alterations in squamous cervical cancers in TCGA. Mutations of the gene occur in equal frequencies in 3q26 amplified and non-amplified cases, suggesting that *PIK3CA* mutations are non-redundant with gene amplifications. This is corroborated by the fact that several cases in TCGA with *PIK3CA* mutations but without concomitant amplification display increased expression of *PIK3CA* at the mRNA level.

PI3K inhibitors are currently available and have obtained regulatory approval for ER-positive, HER2-negative breast cancers bearing *PIK3CA* mutations [11]. The PI3Kα specific inhibitor alpelisib was studied in a phase I study in various solid tumors with *PIK3CA* mutations [29]. Interestingly, among the five cervical cancer patients included, three patients obtained a partial response. Combinations of PI3K inhibitors with other targeted therapies could be of interest for further development. There is, for example, a suggestion from preclinical studies that *PIK3CA* mutations may sensitize cervical cancer cells to PARP inhibitors [30]. High risk HPV strains associated with cervical cancer promote defects in homologous recombination in cervical cancer cells [31]. The HPV oncoproteins E6 and E7 sequester protein RAD51 away from double strand lesions and significantly impair the ability of the cell to repair double strand breaks. Activity of alpelisib with olaparib has been observed in platinum refractory pretreated ovarian cancer patients with a response rate of 36% [32]. The combination would be a rational therapy to be investigated in cervical cancers with DDR defects derived from the presence of viral oncogenes and mutations of genes encoding for DDR proteins, which as shown here are present in a significant minority of squamous cervical carcinomas, mostly with *PIK3CA* mutations.

Increased TMB has been arising as a marker for response to immunotherapy with PD-1/PD-L1 and CTLA-4 inhibitors [33,34]. The PD-1 inhibitor pembrolizumab has obtained accelerated approval in patients with metastatic PD-L1 positive (combined positive score ≥ 1%) cervical cancer who had progressed on chemotherapy [35]. The great majority (83.7%) of patients had PD-L1 positive cancers. The response rate was 14.6%, and thus predictive biomarkers beyond PD-L1 are needed. It would be interesting to clarify whether *PIK3CA* mutations, associated with higher TMB, could be such a biomarker. However, given the low response rates with PD-1 inhibitor immunotherapy therapy in cervical cancer that have also been confirmed in an early nivolumab trial, combination therapies are the most promising and preferable development strategy [36]. In this regard, the combination of nivolumab with ipilimumab has produced response rates between 32% and 46%, depending on the schedule, in a phase I/II multi-cohort trial [37]. Trials of combinations of PD-1 inhibitors with other targeted therapies such as PI3K inhibitors, guided by molecular markers such as *PIK3CA* mutations, should be considered as one of the next steps of cervical cancer therapeutics. An advantage of the combination of PI3K inhibitors with PD-1/PD-L1 is the non-overlapping adverse effect profile of kinase inhibitors with immunotherapeutics. In addition, with a better biomarker targeting, such combinations may increase the sub-sets of patients who derive benefit, without a decrease in quality of life. The adverse effect profile of combinations of targeted therapies will be a key factor in their development especially in the metastatic setting, given the importance of quality of life maintenance in these patients.

## Data Availability

No additional data are available.
